# A Well-Structured Follow-Up Program is Required after Recovery from Coronavirus Disease 2019 (COVID-19); Release from Quarantine is Not the End of Treatment

**DOI:** 10.3390/jcm10112329

**Published:** 2021-05-26

**Authors:** Keun-Mi Lee, Hae-Jin Ko, Geon Ho Lee, A-Sol Kim, Dong-Wook Lee

**Affiliations:** 1 Yeungnam University Medical Center, Department of Family Medicine, School of Medicine, Yeungnam University, Daegu 42415, Korea; kmlee@yu.ac.kr; 2 Department of Family Medicine, School of Medicine, Kyungpook National University, Kyungpook National University Hospital, Daegu 41944, Korea; 3 Department of Family Medicine, Daegu Catholic University School of Medicine, Daegu 42472, Korea; totoslee@cu.ac.kr; 4 Department of Family Medicine, School of Medicine, Kyungpook National University, Kyungpook National University Chilgok Hospital, Deagu 41404, Korea; deepai@knu.ac.kr; 5 Department of Family Medicine, Dongguk University Gyeongju Hospital, Gyeongju 38067, Korea; fmleedu@dongguk.ac.kr

**Keywords:** COVID-19, consultation, comprehensive health care, mental health, pandemics

## Abstract

During the Coronavirus Infection Disease-19 (COVID-19) pandemic, the number of patients released from quarantine is exceeding the number of newly diagnosed cases. This study is a retrospective cohort study in which consultation data were collected from a COVID-19 follow-up health consultation program. The studied population was selected from patients who recovered after quarantine and treatment for COVID-19 in Daegu City and in Gyeongsangbukdo province, Korea, from March to June 2020. The healthcare providers comprised 20 family-medicine specialists who consulted and educated the patients through phone calls in accordance with structured guidelines. Physical and mental status before and after recovery were compared among patients who received a single consultation and those who received two or more consultations. A total of 1604 subjects were selected for the final analysis. Of these, 1145 (71.4%) had one consultation and 459 (28.6%) had two or more. The group that had two or more consultations reported significantly more physical symptoms, more psychological symptoms (including depression), and more psychological stress. Multivariate forward selection logistic regression analysis showed that re-confirmed cases of COVID-19, physical symptoms after quarantine, feelings of depression, and psychological stress had a significant effect on the number of consultations received. In conclusion, COVID-19 has various physical and mental sequelae after discharge from quarantine. Therefore, a well-structured follow-up program is needed after recovery.

## 1. Introduction

Coronavirus Infectious Disease-19 (hereafter referred to as COVID-19), is a respiratory syndrome caused by the SARS-CoV-2 virus that began with an outbreak in Wuhan City, China, in December 2019; since then, it has spread rapidly worldwide [1,2]. In Korea, the 31st patient with COVID-19 was confirmed in Daegu City on 19 February 2020; after that, the disease spread nationwide and prompted the Korean government to raise the infectious disease crisis alert to “serious” on 23 February 2020 [1]. On 12 March, the WHO declared COVID-19 to be a pandemic [3]. COVID-19 has a high basic reproduction number (R0) of 1.9–6.5; however, ~81% of patients are asymptomatic or have mild symptoms [4]. The most common symptoms are fever, fatigue, and a dry cough, although a considerable number of patients report anosmia (loss of the sense of smell) [5].

At the time of writing, Korea has reported >70,000 confirmed cases and >100 million infections; more than 2 million deaths have been reported worldwide and the numbers continue to rise. The number of confirmed cases and the number of people released from quarantine after full recovery is also increasing rapidly [1]. According to the Center for Disease Control and Prevention, release from quarantine is allowed under the following circumstances: absence of fever without the need for antipyretic drugs; improved clinical symptoms for a minimum period of 72 h at 10 days post-onset; and two negative COVID-19 (polymerase chain reaction, (PCR)) tests with an interval of at least 24 h [1]. Since March 2020, when the number of confirmed cases in Korea increased rapidly, the number of patients that recovered began to rise; as of 29 January 2021, the number of recovered patients had reached 66,503, which was seven times that in quarantine [1].

Patients who recover from an infectious disease may experience several sequelae. For example, severe acute respiratory syndrome (SARS) was prevalent in China from 2002 to 2004, and those who recovered had significant psychological problems lasting up to three months post-discharge. Problems included a marked deterioration in quality of life, post-traumatic stress disorder, depression, and anxiety [6]. Furthermore, 44.1% of patients complained of post-traumatic stress disorder even at four months post-discharge [7]. For patients hospitalized in an intensive care unit (ICU) due to severe disease, physical quality of life functions were significantly affected [6], Similarly, ~36% of patients with Middle East Respiratory Syndrome (MERS) reported sequelae such as pulmonary fibrosis even after successful discharge [8]. Therefore, data from patients who recovered from SARS and MERS suggest that COVID-19 is highly likely to have physical or psychological sequelae; indeed, COVID-19 leads to additional health problems such as respiratory symptoms, cognitive impairment, anxiety, depressive symptoms, insomnia, denial, anger, and post-traumatic stress [9,10]. Thus, patient care with psychological support after discharge is an important factor to consider.

An unusual aspect of COVID-19 is that people released from quarantine can test positive again. In China, several such cases have been reported [11,12]. In Korea, many people tested positive for COVID-19 within a short period after viral clearance. The Central Clinical Committee regards this phenomenon as being caused by genetic material from the remaining “dead virus” rather than by a live virus [13]. However, because the period of re-confirmation after a negative virus PCR test varies from 4 to 17 days [12], and re-infection, but not re-confirmation, after recovery is possible [14], there is no question that patients need appropriate care and screening for the recurrence of symptoms, even after discharge.

However, unlike programs designed to increase diagnosis and survival rates, care programs for recovered COVID-19 patients returning to the community are insufficient. Daegu Metropolitan city and Gyeongsangbukdo province had >75% of confirmed COVID-19 cases in Korea; this is because the virus spread via some local religious groups from February to April 2020, the initial period of disease spread in Korea. As mentioned above, the number of patients released from quarantine exceeds the number of new diagnoses; therefore, the proportion of recovered patients in Daegu city and Gyeongsangbukdo province was the highest in Korea [1]. Thus, the Daegu–Gyeongsangbukdo branch of the Korean Family Medicine Association developed a follow-up health consultation program for patients that recovered from COVID-19: 20 family-medicine faculties, in co-operation with Daegu and the Daegu Medical Association, volunteered to begin providing consultations for those who agreed to participate in the program. To the best of our knowledge, this program is the first of its kind in the world. This study investigated the need for, and the results of, a well-structured follow-up program for people who recovered from COVID-19.

## 2. Materials and Methods

### 2.1. Study Design and Subjects

This study is a retrospective cohort study in which consultation data were collected from the COVID-19 follow-up health consultation program and analyzed retrospectively. The program for recovered patients started on 9 March and ended on 5 June 2020. During this period, all patients who had been released from quarantine in Gyeongsangbukdo province were asked to participate in the consultation program in Daegu [15]. As of the date of approval of the research protocol, 1679 (26.9%) of 6247 recovered patients agreed to participate.

A diagnosis of COVID-19 was based upon the isolation of the SARS-CoV-2 virus from a nasopharyngeal sample or detection of a specific gene in a PCR test. A recovered patient was defined as an individual who had met the following clinical and test criteria: absence of fever, improvement in clinical symptoms without the need for antipyretic drugs, and negative results from two PCR tests conducted with an interval of at least 24 h [1]. Subjects who (i) withdrew consent for consultation, (ii) did not respond or could not be contacted by phone, and (iii) were under 18 years-of-age and did not have consent from the primary guardian were excluded. The study complied with the tenets of the Helsinki Declaration and was approved by the Institutional Review Board of the local hospital (protocol No. YUMC 2020-04-112).

Selected subjects were classified into two groups in accordance with the total number of consultations received during the study period: those who required or requested one consultation and those who required or requested more than one consultation.

### 2.2. Methods

#### 2.2.1. A Follow-Up Health Consultation Program after Recovery from COVID-19

The follow-up health consultation program for recovered patients was developed by the Daegu-Gyeongsangbukdo branch of the Korean Family Medicine Association in co-operation with Metropolitan Daegu and the Daegu Medical Association to encourage the provision of health care for patients released from quarantine. The program is described in detail elsewhere [15]. The professional healthcare providers comprised 20 family-medicine specialists who were willing to volunteer for the program; these providers undertook consultations in accordance with structured guidelines published by the Daegu-Gyeongsangbukdo branch of the Korean Family Medicine Association. Using a mobile phone dedicated to individual patient consultations, they called all patients who agreed to participate and asked them about their physical and mental health status. Moreover, they provided emotional support, such as encouragement and reassurance, along with health education to minimize the risk of spreading the virus in the community and to make patients aware of the possibility of reactivation or re-infection of the disease. Each patient was able to call the cellphone of the doctor in charge at any time; if the phone was not answered, the patient received a call-back. In situations requiring further evaluation and treatment, doctors provided medical advice to enable the patient to visit a medical institution or hospital promptly. Patients were provided with a guide for self-care after discharge, the aim of which was to educate them about COVID-19.

#### 2.2.2. General Characteristics of the Subjects and their Symptoms before Release from Quarantine

The general characteristics of the selected subjects included sex, age, underlying disease (past history), and drug use. Information related to COVID-19 included the dates of confirmation, hospitalization, and discharge, the status of hospitalization (inpatient facility or medical institution), and any period in an ICU. For those released from self-quarantine without having to be admitted to a medical institution, the date of diagnosis and the date of release from quarantine were recorded. Moreover, the primary clinical symptoms experienced during the 7 days prior to diagnosis were noted. Clinical symptoms included fever, cough, excess sputum, sore throat, rhinorrhea, nasal congestion, dysosmia, anosmia, dysgeusia, pressure or discomfort in the chest, dyspnea, myalgia, headache, fatigue and malaise, diarrhea, and abdominal discomfort. Asymptomatic cases were also recorded.

#### 2.2.3. Evaluation of Physical Condition after Release from Quarantine

To evaluate the physical condition of patients released from quarantine, symptoms suggestive of recurrence were verified such as shortness of breath, a clinically possible sequela, cough, olfactory abnormality, and headache. Moreover, patients were asked to rate their general physical condition after release from quarantine using a five-point Likert scale: “much better”, “slightly better”, “similar”, “slightly worse”, and “much worse”.

#### 2.2.4. Evaluation of Psychological Status after Release from Quarantine

Psychological status after release from quarantine was verified with respect to anxiety, depression, insomnia, and mental stress. For anxiety and depression, questions to which responses were given on a four-point Likert scale: “never”, “rarely”, “sometimes”, and “often”. The criterion for “never” was the absence of any of the above symptoms. “Rarely” to “often” meant that symptoms affected daily life. Insomnia was rated on a three-point Likert scale: “never”, “sometimes”, and “often”; “often” means that it occurs so often that it affects daily life. Response to stress was rated on a five-point Likert scale: “never”, “very rarely”, “sometimes”, “often”, and “very often”.

#### 2.2.5. Evaluation of Family Relationships after Release from Quarantine

To evaluate changes in family relationships before and after COVID-19 infection, responses were rated on a three-point scale: “closer”, “no change”, and “more distant”.

#### 2.2.6. Positive Test after COVID-19 Viral Clearance

Subgroup analysis was conducted for patients that tested positive again during the consultation period to examine the presence of clinical factors associated with this phenomenon.

### 2.3. Statistical Analysis

Data were analyzed using an independent *t*-test, Pearson’s Chi-square test, and Fisher’s exact test to assess differences between those who received only one consultation and those who received two or more consultations. To confirm factors associated with the number of consultations, multivariate logistic regression analysis with the forward selection method (selection criterion: *p* < 0.05) was applied, using the demographic and clinical characteristics of the subjects as independent variables. All statistical analyses were performed using IBM SPSS statistics version 25.0 software (IBM Corp., Armonk, NY, USA). A value of *p* < 0.05 was considered statistically significant.

## 3. Results

### 3.1. The Participants in the Health Consultation Program

During the study period, 1679 recovered patients agreed to participate. Later, 72 subjects withdrew consent or refused to receive a consultation, and three were excluded for duplicate registration or registration errors; therefore, a total of 1604 subjects were selected for final analysis. Of these, 1145 (71.4%) completed the program with only one consultation, and 459 (28.6%) required two or more consultations. The average number of consultations was 1.38 ± 0.79, and each patient was offered a maximum number of 13 consultations.

### 3.2. General Characteristics of the Subjects, and Symptoms Prior to Release from Quarantine

The mean age of all subjects was 43.62 years, and 33% were male. Note that 1.7% had symptoms severe enough to require inpatient treatment in an ICU, whereas 22.8% had more than one underlying disease, the most common of which was hypertension (12.8%). Up to 7 days before a positive diagnosis of COVID-19, 74.6% of participants had symptoms, the most common being cough, excess sputum, fever, and myalgia. Although 25.4% of the subjects were asymptomatic, COVID-19 was confirmed through screening tests that were conducted for reasons such as a close contact with a positive case.

Compared with the group that received only one consultation, the group that received two or more had more ICU hospitalizations and more symptoms, including cough/sputum, chest tightness or shortness of breath, and fatigue or lethargy. There was no significant difference between the two groups with respect to age, sex, and underlying disease (Table 1).

There were no significant differences in the number of consultations or the characteristics of assigned patients between the 20 physicians (Table S1).

### 3.3. Physical and Psychological Status and Family Relationships after Release from Quarantine

Table 2 shows the physical and psychological status of the subjects after release from quarantine. Overall, 27% said they had physical symptoms after release. The most common were cough/sputum, olfactory/taste disorders, sore throat, fatigue and weakness, and chest tightness. Moreover, 21.8% said their overall health status seemed worse; 33% reported anxiety; 19.3% reported depression; 23.5% reported insomnia; and 53.2% reported stress. In addition, 5.4% reported a deterioration in family relationships.

After release from quarantine, the group that received two or more consultations had more physical symptoms than the group that received a single consultation (23.7% vs. 35.3%, *p* < 0.001). Symptoms included cough, fatigue, sore throat, and dysosmia/dysgeusia.

Compared with the group that received a single consultation, the group that received two or more consultations had more psychological symptoms, including anxiety (29.0% vs. 42.9%, *p* < 0.001) and depression (15.3% vs. 29.8%, *p* < 0.001). This included mild, moderate, or severe anxiety; and a mild, moderate or severe depressive mood. In addition, the group that received two or more consultations experienced more psychological stress: mild, moderate, or severe. There was no significant difference between the two groups with respect to changes in overall physical health status or family relationships.

### 3.4. Factors Affecting the Number of Consultations after Recovery from COVID-19

Multivariate forward-selection logistic regression analysis was conducted using relevant demographic and clinical characteristics as independent variables to identify factors that led to a requirement for two or more consultations with a doctor. Those with re-confirmed COVID-19 (adjusted odds ratio (aOR): 6.703), had more physical symptoms after quarantine (aOR: 1.558), felt depressed (aOR: 1.668 for mild depression, 1.922 for moderate depression), or felt psychological stress (aOR: 1.418 for rarely stressed, 2.029 for frequently stressed); and required more consultations (Table 3).

### 3.5. Positive Cases after Clearance of Virus and a Negative Test Result

Subjects with no clinical symptoms were released from quarantine after two negative COVID-19 PCR test results (performed after an interval of 24 h or more). Nevertheless, 35 cases (2.2%) became positive again during the program: 5.7% in the two or more consultations group and 0.8% in the single consultation group (Table 2). Two of these 35 cases (5.7%) were hospitalized in an ICU after the first diagnosis and quarantined for a mean of 31.49 days. Twelve patients (34.3%) were tested again on the doctor’s recommendation or at their own request due to residual symptoms. Twenty-three patients (65.7%) had no symptoms but were confirmed as positive by a mandatory screening test taken before returning to work or health care facilities (Table 4).

## 4. Discussion

This study was the first to conduct and analyze a follow-up health consultation program provided to patients who recovered from COVID-19 and lived in the region of Korea most severely affected by the outbreak. After the COVID-19 pandemic declaration, social distancing was strictly enforced in Korea, and all individuals with a confirmed infection were quarantined in a single hospitalization room until they recovered fully. Patients were discharged if they showed no physical symptoms and had two or more negative PCR tests. However, despite successful recovery, people reported marked physical and psychological sequelae, meaning that they required further medical advice. Moreover, 28.6% of these individuals required several consultations with doctors. Overall, 27% had physical symptoms; 33% had anxiety; 19.3% had depression; 23.5% had sleep problems, and 53.2% had mental stress. In particular, of the subjects that required two or more consultations, the number of re-confirmed cases was significantly higher among those who were hospitalized in the ICU, those who were symptomatic before hospitalization, or those who complained of physical symptoms after release from quarantine. Furthermore, a higher percentage of those who required multiple consultations reported mild anxiety, mild depression, and mild mental stress than those who required only a single consultation. These results strongly suggested that a follow-up health consultation program delivered by medical professionals and psychologists would play an important role in patient care after release from COVID-19 quarantine.

As mentioned above, patients who recovered from SARS and MERS also reported significant psychological problems after discharge [6,7]. However, the level of psychological distress reported for COVID-19 was much higher, principally because SARS-CoV-2 virus has neurological sequelae through both neuroinvasive and neurovirulent mechanisms. Studies show that SARS-CoV-2 can affect the central nervous system and infiltrate neurons [16]. In addition, unlike SARS or MERS, COVID-19 was declared a pandemic, after which countries implemented strict lockdowns. These lockdowns have had a negative effected on the mental health of the general population, as well as those infected with COVID-19 [17,18]. Also, and most importantly, if a person is infected or has been in close contact with someone who is, he or she has to self-isolate for several weeks. The average period of quarantine in this study was 26 days; patients spent ~4 weeks in an isolated space (hospital room or community treatment center). In particular, psychological distress was severe because patients were allowed no direct contact with others. The mental support provided by professional healthcare providers and psychologists was very important to patients isolated under such conditions. This notion is supported by the results of multivariate analysis, which identified depressive mood and psychological stress as factors that affected the decision to request more consultations.

COVID-19 has a spectrum of clinical symptoms ranging from asymptomatic to severe pneumonia and death [19]. Clinically, the most common symptoms are fever, cough, fatigue, and dyspnea (in that order) [20]. Analysis of 10,237 Korean patients with confirmed COVID-19 revealed that 62% were asymptomatic [21]. Among 40 confirmed patients in a city in Korea, 5% were asymptomatic [22], whereas in an Italian study it was 42.5% [23]. The proportion of asymptomatic patients infected with COVID-19 is thought to be ~40–45% [24]. Among the 1604 subjects examined in this study, 408 (25.4%) were asymptomatic. Common symptoms at diagnosis were cough, fever, myalgia, and dysosmia (in that order), a finding that was somewhat different from that reported in previous studies, probably because the numbers in the study cohorts varied from study to study. In addition, the policies and criteria used for screening tests to identify asymptomatic infections differed from country to country. Moreover, because this study included only those who agreed to participate in the consultation program, there was a possibility that relatively more symptomatic patients joined the program. We thought it interesting that, out of 408 subjects who stated that they were asymptomatic at the time of diagnosis, 29 (7.1%, data not shown) said that they had residual physical symptoms when asked. From March to April 2020, the outbreak in Daegu spread rapidly among a certain religious group [25]. They might not have answered honestly due to a fear of social stigma if they were infected because all their movements and relationships would be exposed [26]. Thus, they might have said they had no symptoms when in fact they did.

In general, according to WHO guidelines, patients can be hospitalized in a ICU if they show severe symptoms, ranging from severe disease to acute respiratory distress syndrome and sepsis [27]. Older age, diabetes, higher body temperature, and lower peripheral oxygen saturation all increase the possibility of ICU hospitalization [28]. Here, we found that subjects diagnosed with COVID-19 based on their symptoms, or those admitted to the ICU owing to the severity of COVID-19 symptoms, experienced more residual symptoms after discharge than those who were asymptomatic. This outcome is plausible because polyneuropathy, myopathy, and reduced pulmonary function are highly likely in patients who have severe disease [29,30,31]. Therefore, patients with symptomatic or severe COVID-19 infection required a more detailed follow-up program that allows close observation, even after discharge.

We found that 35 patients (2.2%) had re-confirmed COVID-19 after release from quarantine, and this group had the greatest effect on the number of consultations (aOR, 6.703). According to a report by the Korea Centers for Disease Control and Prevention, 292 re-confirmed cases (3.3% of 8922) were reported up until 29 April 2020; these were ascribed not to reactivation or re-infection, but to detection of genetic material from “dead virus” [13]. Similarly, the incidence of re-confirmed COVID-19 RT–PCR results in Italy was 13.7% on Day 14 post-discharge and 14.7% on Day 41 post-discharge [32]. The figure for China was 19% [33]. Here, 12 patients (34.3%) had re-confirmed infection after taking a test on the recommendation of a doctor during the follow-up consultation program because these patients showed residual symptoms that were considered minor but clinically important. Similar results were found in China, where 28% of re-confirmed patients complained of mild symptoms [33]. Re-confirmed cases are much less likely to transmit the disease than those with active infections [13]; however, the viral load is related to the clinical severity of COVID-19 [34], and concerns of infections driven by re-confirmed cases are increasing [14,35,36]. Therefore, physicians must verify whether recovered patients show symptoms; even mild symptoms. Accordingly, a well-structured follow-up program after recovery from COVID-19 (provided by healthcare professionals) will be an effective way to decide whether a patient requires another RT–PCR test.

This study has the following limitations. First, the results cannot be generalized over the general population because it was conducted on an ethnically homogeneous population in a single city. Second, because only patients who agreed to participate in the consultation program were enrolled, there is a possibility of selection bias and overestimation of the results. Third, even though the 20 consulting doctors had the same specialty, they are not professional psychiatrists, so the quality and quantity of consultation may vary. However, a general guideline was provided and a web-based communication site was set up to share information with the consulting doctors to minimize inter-physician variation. Fourth, standardized assessment tools were not applied to evaluate physical and psychological status. Further prospective cohort studies using validated assessment tools are needed.

Despite these limitations, this study has certain strengths. It is the first in which professional healthcare providers implemented a patient management program after discharge; moreover, because Daegu was in the region with the highest number of confirmed cases from February to April 2020, the results could be taken as a reflection of the Korean population during the early COVID-19 pandemic period.

## 5. Conclusions

This study showed that COVID-19 has various physical and mental sequelae after discharge, and that patients require follow-up medical consultations. COVID-19 is still prevalent and is still having adverse economic and social effects worldwide. It is necessary to develop and publicize a systematic follow-up care program to provide comprehensive health care for patients recovering from this highly contagious disease.

## Figures and Tables

**Table 1:** Clinical characteristics of the subjects during and before infection with COVID-19.

	Total (*n* = 1604)	No. of Consultations		*p* *
		Once (*n* = 1145)	Twice or More (*n* = 459)	
No. of consultations	1.38 ± 0.79	1.00	2.34 ± 0.95	<0.001
Age	43.62 ± 16.32	43.46 ± 16.40	44.02 ± 16.13	0.765
Sex, male	530 (33)	381 (33.3)	149 (32.5)	0.754
Quarantine period (days)	26.16 ± 9.99	26.32 ± 9.85	25.71 ± 10.33	0.376
ICU admission	28 (1.7)	15 (1.3)	13 (2.8)	0.035
Underlying disease	366 (22.8)	265 (23.1)	101 (22.0)	0.623
Hypertension	206 (12.8)	146 (12.8)	60 (13.1)	0.862
Diabetes	95 (5.9)	68 (5.9)	27 (5.9)	0.965
Dyslipidemia	25 (1.6)	20 (1.7)	5 (1.1)	0.337
Heart disease	26 (1.6)	16 (1.4)	10 (2.2)	0.263
Pulmonary disease	27 (1.7)	18 (1.6)	9 (2.0)	0.584
Allergic disease	29 (1.8)	22 (1.9)	7 (1.5)	0.590
Thyroid disease	12 (0.7)	8 (0.7)	4 (0.9)	0.751 ^†^
Kidney disease	4 (0.2)	3 (0.3)	1 (0.2)	1.000 ^†^
Any malignancies	20 (1.2)	17 (1.5)	3 (0.7)	0.175
Liver disease	12 (0.7)	7 (0.6)	5 (1.1)	0.341 ^†^
Neurologic disease	17 (1.1)	13 (1.1)	4 (0.9)	0.791 ^†^
Presence of symptoms before the diagnosis of COVID-19				
Asymptomatic	408 (25.4)	310 (27.1)	98 (21.4)	0.017
Any symptom	1196 (74.6)	835 (72.9)	361 (78.6)	
Fever	342 (21.3)	235 (20.5)	107 (23.3)	0.218
Cough, sputum	346 (21.6)	232 (20.3)	114 (24.8)	0.044
Sore throat, pharyngitis	180 (11.2)	126 (11.0)	54 (11.8)	0.663
Rhinorrhea, nasal congestion	96 (6.0)	71 (6.2)	25 (5.4)	0.565
Dysosmia, dysgeusia	212 (13.2)	150 (13.1)	62 (13.5)	0.828
Chest discomfort, dyspnea	73 (4.6)	40 (3.5)	33 (7.2)	0.001
Myalgia	308 (19.2)	223 (19.5)	85 (18.5)	0.660
Headache	122 (7.6)	86 (7.5)	36 (7.8)	0.821
Fatigue, malaise, lethargy	28 (1.7)	15 (1.3)	13 (2.8)	0.035
Diarrhea, abdominal discomfort	61 (3.8)	39 (3.4)	22 (4.8)	0.189

Data are presented as the mean ± standard deviation or as number (%). * Independent t-test for continuous variables, and Pearson’s Chi-square test or ^†^ Fisher’s exact test for discrete variables. COVID-19, coronavirus infectious disease-19; ICU, intensive care unit.

**Table 2:** Physical and psychological status after recovery from COVID-19.

	Total (*n* = 1604)	No. of Consultations		*p* *
		Once (*n* = 1145)	Twice or More (*n* = 459)	
Physical symptoms after recovering from COVID-19				
Asymptomatic	1171 (73.0)	874 (76.3)	297 (64.7)	<0.001
Any symptom	433 (27.0)	271 (23.7)	162 (35.3)	<0.001
Fever	10 (0.6)	5 (0.4)	5 (1.1)	
Cough, sputum	174 (10.9)	116 (10.1)	58 (12.6)	
Sore throat, pharyngitis	40 (2.5)	23 (2.0)	17 (3.7)	
Rhinorrhea, nasal congestion	33 (2.1)	26 (2.3)	7 (1.5)	
Dysosmia, dysgeusia	44 (2.7)	27 (2.4)	17 (3.7)	
Chest discomfort, dyspnea	38 (2.4)	23 (2.0)	15 (3.3)	
Myalgia	23 (1.4)	13 (1.1)	10 (2.2)	
Headache	23 (1.4)	16 (1.4)	7 (1.5)	
Fatigue, malaise, lethargy	40 (2.5)	18 (1.6)	22 (4.8)	
Diarrhea, abdominal discomfort	8 (0.5)	4 (0.3)	4 (0.9)	
General physical status after recovering from COVID-19				
Much better than before	31 (1.9)	25 (2.2)	6 (1.3)	0.057
A little better than before	138 (8.6)	107 (9.3)	31 (6.8)	
Similar	1085 (67.6)	782 (68.3)	303 (66.0)	
A little worse than before	314 (19.6)	208 (18.2)	106 (23.1)	
Much worse than before	36 (2.2)	23 (2.0)	13 (2.8)	
Anxiety after recovering from COVID-19				
Not at all	1075 (67.0)	813 (71.0)	262 (57.1)	<0.001 ^†^
Mild	437 (27.2)	284 (24.8)	153 (33.3)	
Moderate	88 (5.5)	47 (4.1)	41 (8.9)	
Severe	4 (0.2)	1 (0.1)	3 (0.7)	
Depressive mood after recovering from COVID-19				
Not at all	1295 (80.7)	970 (84.7)	325 (70.8)	<0.001 ^†^
Mild	244 (15.2)	143 (12.5)	101 (22.0)	
Moderate	61 (3.8)	30 (2.6)	31 (6.8)	
Severe	4 (0.2)	2 (0.2)	2 (0.4)	
Insomnia after recovering from COVID-19				
Not at all	1227 (76.5)	920 (80.3)	307 (66.9)	<0.001
Occasionally	199 (12.4)	125 (10.9)	74 (16.1)	
Frequently	178 (11.1)	100 (8.7)	78 (17.0)	
Psychological stress after recovering from COVID-19				
Not at all	751 (46.8)	585 (51.1)	166 (36.2)	<0.001
Rarely	417 (26.0)	287 (25.1)	130 (28.3)	
Occasionally	301 (18.8)	203 (17.7)	98 (21.4)	
Frequently	114 (7.1)	60 (5.2)	54 (11.8)	
Very often	21 (1.3)	10 (0.9)	11 (2.4)	
Family relationships after recovering from COVID-19				
Closer than before	97 (6.0)	74 (6.5)	23 (5.0)	0.176
Unchanged	1421 (88.6)	1016 (88.7)	405 (88.2)	
Deteriorated	86 (5.4)	55 (4.8)	31 (6.8)	
Re-positive result of COVID-19 test	35 (2.2)	9 (0.8)	26 (5.7)	<0.001

Data are presented as number (%). * Pearson’s Chi-squared and ^†^ Fisher’s exact tests.

**Table 3:** Factors affecting the decision to request more than two consultations after recovering from COVID-19.

Factors	aOR	95% CI	*p* *
Re-confirmed COVID-19 test	6.703	3.062–14.674	<0.001
Presence of physical symptoms after recovering from COVID-19			
Asymptomatic	1.000		
Any symptom	1.558	1.220–1.989	<0.001
Depressive mood after recovering from COVID-19			
Not at all	1.000		
Mild	1.668	1.202–2.314	0.002
Moderate	1.922	1.034–3.573	0.039
Severe	1.961	0.268–14.374	0.508
Psychological stress after recovering from COVID-19			
Not at all	1.000		
Rarely	1.418	1.072–1.874	0.014
Occasionally	1.182	0.843–1.658	0.331
Frequently	2.029	1.248–3.299	0.004
Very often	1.963	0.751–5.130	0.169

* Multivariate forward selection logistic regression analysis using frequent (≥2) consultation as a dependent variable. aOR, adjusted odds ratio; CI, confidence interval.

**Table 4:** Clinical characteristics of the patients with re-confirmed COVID-19 after viral clearance evidenced by a negative test result.

	Characteristics of Re-Confirmed Cases (*n* = 35)
Age, years	49.11 ± 15.35
Sex, male	8 (22.9)
Quarantine period (days)	31.49 ± 10.86
ICU admission	2 (5.7)
Underlying disease	8 (22.9)
Hypertension	7 (20.0)
Dyslipidemia	1 (2.9) *
Neurologic disease	1 (2.9) ^†^
Purpose of COVID-19 re-test	
For their remaining symptoms	12 (34.3)
For returning to work or health care facilities	23 (65.7)

Data are presented as the mean ± standard deviation or as number (%). * One had both hypertension and dyslipidemia. ^†^ Meningitis. ICU, intensive care unit.

## Data Availability

Please contact author for data requests.

## Supplementary Materials

The following are available online at https://www.mdpi.com/article/10.3390/jcm10112329/s1, Table S1: The characteristics of the subjects according to the physicians.
