# Supplementary material for: A Well-Structured Follow-Up Program is Required after Recovery from Coronavirus Disease 2019 (COVID-19); Release from Quarantine is Not the End of Treatment
Source: J Clin Med. 2021 May 26;10(11):2329. doi: 10.3390/jcm10112329 (PMC8198537; doi:10.3390/jcm10112329)
Supplement: Supplementary file 1 [file jcm-10-02329-s001.zip › jcm-1189572-supplementary.pdf]

Table S1. The characteristics of the subjects according to the physicians.

Physician	No. of assigned patients	No. of consultations	Age of assigned patients (years)	Sex of assigned patients (male)
#1	84	1.37 ± 0.66	43.07 ± 16.81	25 (29.8%)
#2	81	1.41 ± 0.67	41.64 ± 16.02	26 (32.1%)
#3	82	1.66 ± 1.17	43.55 ± 16.74	30 (36.6%)
#4	80	1.44 ± 0.69	44.38 ± 17.10	25 (31.3%)
#5	81	1.44 ± 0.69	46.01 ± 16.03	23 (28.4%)
#6	80	1.39 ± 0.76	42.83 ± 16.17	24 (30.0%)
#7	81	1.31 ± 0.65	44.91 ± 15.34	24 (29.6%)
#8	80	1.31 ± 0.59	43.98 ± 15.87	27 (33.8%)
#9	81	1.37 ± 0.60	46.62 ± 17.17	27 (33.3%)
#10	78	1.33 ± 0.57	42.47 ± 16.73	22 (28.2%)
#11	80	1.46 ±1.45	41.41 ± 15.27	29 (36.3%)
#12	79	1.37 ± 0.62	40.84 ± 15.74	23 (29.1%)
#13	84	1.26 ± 0.54	44.02 ± 15.94	25 (29.8%)
#14	79	1.30 ± 0.54	45.10 ± 16.84	26 (32.9%)
#15	77	1.36 ± 0.84	42.25 ± 16.80	20 (26.0%)
#16	79	1.27 ± 0.50	47.39 ±16.35	25 (31.6%)
#17	80	1.34 ± 0.53	44.63 ± 13.115	45 (56.3%)
#18	78	1.51 ± 1.44	41.29 ± 17.25	22 (28.2%)
#19	81	1.42 ± 0.74	42.85 ± 16.55	34 (42.0%)
#20	79	1.33 ± 0.64	42.96 ± 18.07	28 (35.4%)
p^*		0.343	0.492	0.052

*ANOVA for continuous variables, and Pearson's Chi-square test for discrete variables.
